# CTRP12: A Novel Biomarker in Predicting In‐Stent Restenosis Occurrence

**DOI:** 10.1002/clc.70268

**Published:** 2026-01-31

**Authors:** Ya Nie, Wanmei Song

**Affiliations:** ^1^ Department of Cardiovascular Department Longhui County People's Hospital, Longhui Shaoyang China

**Keywords:** biomarker, coronary artery disease, in‐stent restenosis

Dear Editor,

We read with great interest the recent study entitled *“Changes of Serum CTRP12 in Patients With Coronary Artery Disease After the Treatment of Percutaneous Coronary Intervention and Its Relationship With In‐Stent Restenosis”*. The authors provide compelling evidence supporting C1q/tumor necrosis factor–related protein 12 (CTRP12) as a promising biomarker for both coronary artery disease (CAD) severity and the prediction of in‐stent restenosis (ISR) after PCI [1]. We believe this work adds important translational value to the field of interventional cardiology. Several aspects underscore why CTRP12 may be regarded as a novel and clinically meaningful biomarker.

## 1 First, CTRP12 Reflects Both Disease Burden and Inflammatory Status

The demonstrated negative correlations between serum CTRP12 levels and the Gensini score as well as hs‐CRP highlight its close association with coronary atherosclerotic severity and systemic inflammation. Unlike traditional biomarkers that primarily capture either ischemic burden or inflammation, CTRP12 appears to integrate both structural disease severity and inflammatory activity, two central drivers of ISR pathophysiology [2].

## 2 Second, CTRP12 Exhibits Dynamic Responsiveness to PCI‐Related Vascular Injury

The observed acute decline in CTRP12 levels at 24 h after PCI, followed by partial recovery at 72 h, suggests that CTRP12 is sensitive to procedural vascular trauma and early inflammatory responses induced by stent implantation. This dynamic behavior distinguishes CTRP12 from static baseline biomarkers and supports its role as a real‐time indicator of vascular healing or maladaptive repair processes that predispose to restenosis [3].

## 3 Third, CTRP12 Demonstrates Predictive Value for ISR Beyond Baseline Assessment

Notably, CTRP12 levels at 24 h post‐PCI showed the highest predictive accuracy for ISR, and patients who developed ISR consistently exhibited lower CTRP12 levels at all measured time points. This temporal predictive capacity positions CTRP12 as a potentially actionable biomarker, enabling early risk stratification and closer surveillance or intensified therapy in high‐risk patients.

In summary, CTRP12 emerges as a novel biomarker that links inflammation, coronary disease severity, and post‐PCI vascular response. Future prospective studies and mechanistic investigations are warranted to validate its clinical utility and to explore whether modulation of CTRP12‐related pathways could represent a therapeutic target for preventing ISR.

## Conflicts of Interest

The authors declare no conflicts of interest.

## Data Availability

The data supporting this study are available upon reasonable request from the corresponding author.
